# Fabrication of a Poly(3-octylthiophene-2,5-diyl) Electrochemiluminescence Device Assisted by Perylene

**DOI:** 10.3390/ma6051704

**Published:** 2013-04-29

**Authors:** Tatsuya Daimon, Eisuke Nihei

**Affiliations:** Graduate School of Science and Technology, Keio University, 3-14-1, Hiyoshi, Kohoku-ku, Yokohama 223-8522, Japan; E-Mail: eisuke@appi.keio.ac.jp

**Keywords:** electrochemiluminescence, poly(3-octylthiophene-2,5-diyl), perylene, light-emitting assistance, liquid type light-emitting device

## Abstract

In this study, we report the light-emitting assistance effect of perylene on a polymer electrochemiluminescence (ECL) device using poly(3-octylthiophene-2,5-diyl) (P3OT). An ECL device is a liquid type self-luminous device with a simple structure, and can be fabricated by a relatively easy procedure. Significant improvement in luminescence properties was confirmed when 1.0 wt % perylene was added to the ECL device using 3.0 wt % P3OT. Improvements of about 12 times of the maximum luminescence intensity and about 23 times of the light-emitting time ratio compared with that of a P3OT ECL device were obtained. We conclude that the light-emitting assistance of perylene is achieved by perylene radical ions shuttling electrons to P3OT while they are moving around in the emitting solution. The light-emitting assistance effect of perylene was also confirmed when poly(3-dodecylthiophene-2,5-diyl), which has almost identical electrochemical and photophysical characteristics to those of P3OT, was used instead of P3OT.

## 1. Introduction

In recent years, light-emitting phenomena such as electroluminescence (EL) in organic materials have attracted a growing scientific and commercial interest for their potential application in light-emitting devices such as flat-panel displays and lighting [1,2,3]. Electrochemiluminescence (ECL) treated in this study is also one of such light-emitting phenomena. It is based on energetic electron transfer (redox) reactions of electrochemical species in solution. Namely, luminescent species are oxidized and reduced at the anode and cathode, respectively. Then, radical cations and anions are generated at each electrode. These radical cations and anions move toward the opposite electrode by ion conduction and collide with each other. From the electron transfer that occurs radical cations and anions combination, ground-state and excited-state molecules are generated (in this paper, this phenomenon is called the annihilation reaction), and luminescence is observed from the excited-state molecules [4]. Serious research on ECL has mainly been applied to analysis fields including a biosensor [5,6]. More recently, research related to liquid type self-luminous devices based on ECL has become the subject of active investigation [7,8,9,10].

The ECL device has a very simple structure and usually consists of an emitting solution sandwiched between two electrodes [7]. Therefore, it is free from problems that the EL device faces, such as a complex fabrication procedure and the difficulty in enlarging the device due to the vacuum evaporation process [11]. Moreover, fabrication of the ECL device using a wet process enables the realization of low-cost display applications, and at the same time, the liquid system makes the application as a flexible display promising [8]. Furthermore, the luminescence mechanism of the ECL device makes the drive by voltage of direct-current (DC) as well as alternating-current (AC) possible. When AC voltage is applied, the annihilation reaction between radical cations and anions mainly occurs near electrodes, accompanied by reversal of polarization [9,10].

Although the ECL device seems promising for various features and has the possibility of application for display, its luminescence properties such as short luminescence time and low intensity have become ongoing challenges. Especially the short luminescence time is the main challenge in realizing the application for display. It is thought that the major reason for short luminescence is the termination of redox reactions due to degradation of the emitting material [4]. 

In the past, we thought that the degradation of the emitting material did not occur due to using structurally stable polymer materials, which have advanced studies on polymer ECL devices. Previously, we fabricated two types of polymer ECL device using poly(2,5-dioctylphenylene-ethynylene) and poly(3-octylthiophene-2,5-diyl) (P3OT), and reported their luminescence properties [12,13]. More recently, we have found that the luminescence properties of the P3OT ECL device can be improved by adding perylene. As a consequence, in this study, we investigate the luminescence properties of the P3OT ECL device containing perylene and discuss its luminescence mechanism.

## 2. Results and Discussion

### 2.1. Luminescence Properties of a P3OT ECL Device Containing Perylene 

**Figure 1:**
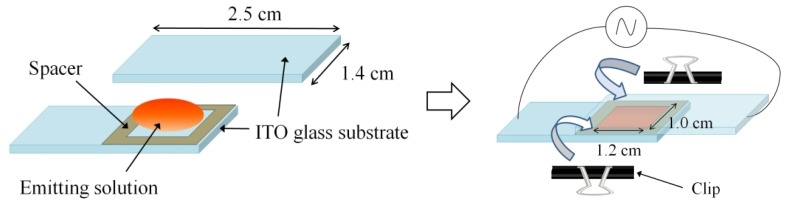
Schematic illustration of the electrochemiluminescence (ECL) device’s structure and fabrication.

The polymer ECL device was fabricated by relatively easy procedures. A schematic illustration of the ECL device structure and fabrication is shown in Figure 1. Two pre-cleaned indium tin oxide (ITO) glass substrates (1.4 × 2.5 cm^2^) were prepared and used as electrodes. Emitting solution is typically prepared by dissolving emitting material and supporting electrolyte in solvent [9]. In this study, emitting solution was prepared by dissolving 3.0 wt % P3OT and 0.3 wt % (0.01 M) tetra-butyl ammonium perchlorate (TBA) in 1,2-dichlorobenzene (DCB), and adding perylene in quantities depending on the experiment. The prepared emitting solution was placed between two electrodes, which were shifted about 1.5 cm in the transverse direction. The upper and lower sides of the electrode were fixed with clips. The active electrode area was 1.0 × 1.2 cm^2^. The distance between electrodes was adjusted by a polyimide film spacer of 12.5 μm. 

ECL was observed by applying an AC voltage of rectangular wave to the polymer ECL device fabricated by the above procedures.

**Figure 2:**
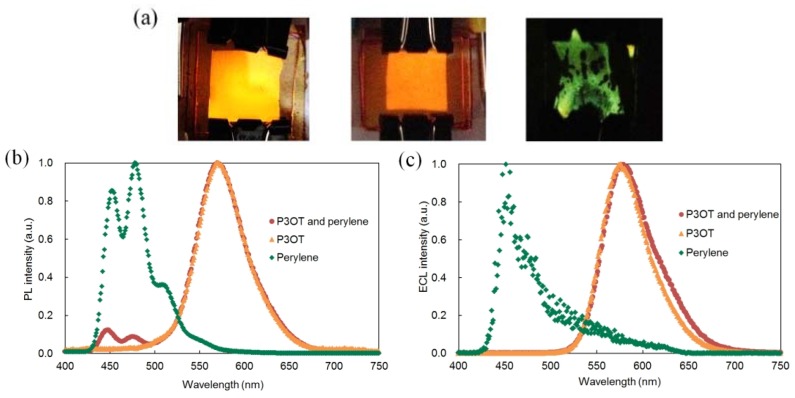
(**a**) Photographs of ECL from the ECL device using: (top) Poly(3-octylthiophene-2,5-diyl) (P3OT) and perylene under application of AC voltage of 5.0 V at 250 Hz; (center) P3OT under the application of AC voltage of 5.0 V at 250 Hz; (bottom) perylene under the application of AC voltage of 12.0 V at 50 Hz. (**b**) Photoluminescence (PL) and (**c**) ECL spectra of each device. Applied voltages are the same as those of (**a**). PL spectra are observed upon excitation with a 350 nm xenon lamp.

Figure 2a shows the appearances of ECL observed from the ECL device using P3OT and perylene (top), P3OT (center), and perylene (bottom), respectively. The P3OT ECL device containing perylene and the P3OT ECL device emitted yellow luminescence, and the perylene ECL device emitted blue-green luminescence. In order to obtain ECL from the perylene ECL device, applying an AC voltage of more than 8.0 V was required. Hence, the electrolysis of the solvent occurred notably, and luminescence was observed only from part of the device. 

The normalized photoluminescence (PL) and ECL spectra of these devices are shown in Figure 2b,c, respectively. The PL spectra were observed upon excitation with a 350 nm xenon lamp, and the ECL spectra were observed by applying AC voltage. 

PL spectrum of the P3OT ECL device containing perylene had a large luminescence maximum at 570 nm and small luminescence maxima at 447 and 476 nm. The large luminescence maximum corresponds well with the luminescence maximum of the P3OT ECL device at 571 nm, and the small luminescence maxima correspond well with the luminescence maxima of perylene ECL device at 453 and 478 nm. This result indicates that the P3OT ECL device containing perylene exhibits PL from two kinds of material, P3OT and perylene, by ultraviolet exposure. In addition, the quenching of perylene in the presence of P3OT was observed. It is considered to be due to the overlap between perylene PL and P3OT absorption spectra in the wavelength band from about 400 to 550 nm.

On the other hand, the ECL spectrum of the P3OT ECL device containing perylene had only one luminescence maximum at 577 nm. This luminescence maximum is identical to that of the P3OT ECL device at 577 nm. It is likely that the non-generation of excited-state perylene molecules is the reason for the disappearance of the luminescence maximum corresponding to that of the perylene ECL device at 452 nm. In fact, luminescence was not observed from the perylene ECL device with the same voltage as in the case of the ECL spectrum measurement of the P3OT ECL device containing perylene (AC voltage of 5.0 V at 250 Hz.). Therefore, ECL from the P3OT ECL device containing perylene is presumed to occur only from excited-state P3OT molecules. Considering the experimental results in which the luminescence properties of the P3OT ECL device were improved by adding perylene, it is predicted that perylene assists ECL of P3OT in some way although perylene itself does not emit luminescence.

**Figure 3:**
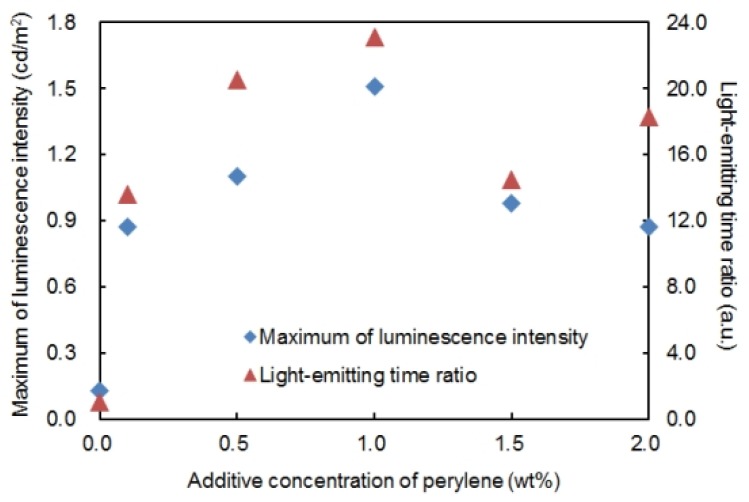
Maximum of luminescence intensity (square blue plots) and light-emitting time ratio (triangle red plots) of the poly(3-octylthiophene-2,5-diyl) electrochemiluminescence device with different additive concentrations of perylene from 0.0 to 2.0 wt %. Applied voltage is AC voltage of 5.0 V at 417 Hz.

For the purpose of investigating quantitatively the light-emitting assistance effect of perylene on the P3OT ECL device, the maximum luminescence intensity and light-emitting time ratio with different additive concentrations of perylene were evaluated. The results are shown in Figure 3. The additive concentration of perylene is on the horizontal axis, and the maximum luminescence intensity (square blue plots) and the light-emitting time ratio (triangle red plots) are on the vertical axis. AC voltage of 5.0 V at 417 Hz was applied. In this paper, the period of time when the ECL device emits luminescence for more than 0.10 cd/m^2^, luminescence intensity is defined as “the light-emitting time”. Moreover, the ratio of the light-emitting time of the target device against the light-emitting time of the P3OT ECL device when an AC voltage of 5.0 V at 417 Hz was applied (90 s) is defined as “the light-emitting time ratio”. If the light-emitting time of the target device was 900 s, for example, the light-emitting time ratio is set to 10.0. In addition, the maximum luminescence intensity is the largest value of luminescence intensity at which the time variation of luminescence intensity was seen.

As shown in Figure 3, an improvement in luminescence properties was confirmed even with the addition of perylene of 0.1 wt %. The luminescence properties of the P3OT ECL device were improved with the increase in the additive concentration of perylene. In particular, the improvement in luminescence properties became most remarkable when 1.0 wt % perylene was added, and at that point, about 12 times improvement of the maximum luminescence intensity and about 23 times improvement of the light-emitting time ratio were obtained. However, when more than 1.0 wt % perylene was added, a decrease in the maximum of luminescence intensity was observed due to the increase in additive concentration of perylene. Such results are probably due to the excess superfluous insoluble perylene in the emitting solution having a negative influence on luminescence. Hence, it is important for maximizing the light-emitting assistance effect of perylene to have a moderate concentration. Based on experimental results, the addition of 1.0 wt % perylene was considered moderate for the ECL device using 3.0 wt % P3OT.

**Figure 4:**
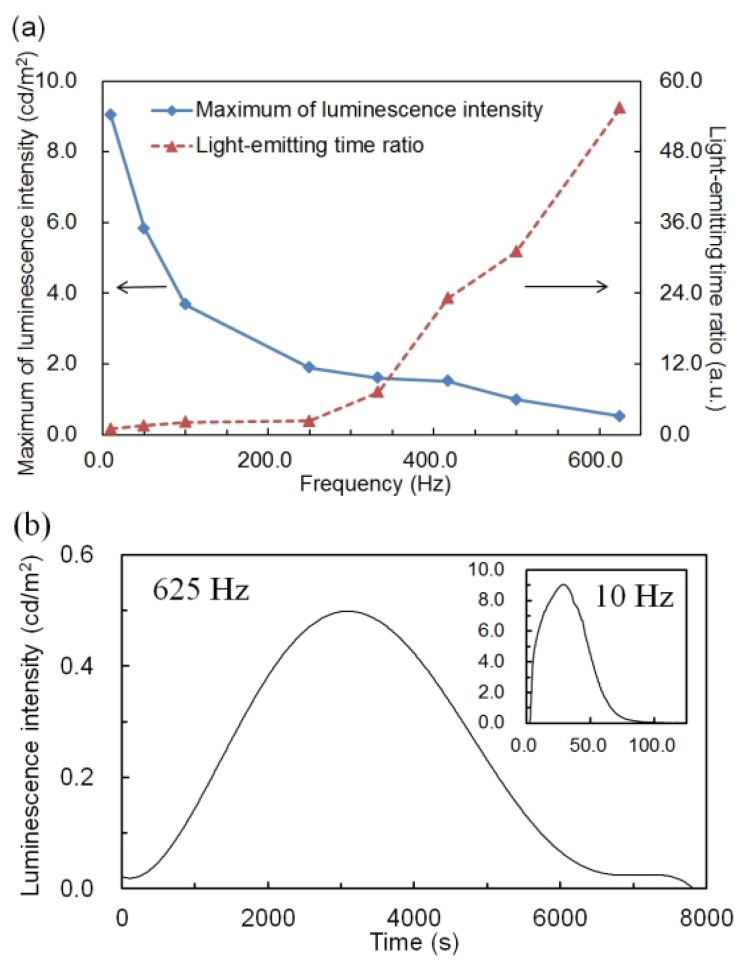
(**a**) Maximum of luminescence intensity (square blue plot and solid line) and light-emitting time ratio (triangle red plot and dashed line) of the poly(3-octylthiophene-2,5-diyl) electrochemiluminescence device with different applied frequencies from 10 to 625 Hz. Applied voltage is AC voltage of 5.0 V and additive concentration of perylene is 1.0 wt %. (**b**) Time variation of luminescence intensity under the application of AC voltage of 5.0 V at 625 Hz and that of 5.0 V at 10 Hz (Inset).

Applied frequency is an important parameter for an AC voltage driven ECL device, similar to the effect of the additive concentration of perylene. Next, the change of light-emitting assistance effect of perylene accompanying the change of applied frequency was evaluated. Figure 4a shows the maximum luminescence intensity and light-emitting time ratio with different applied frequencies. Frequency is on the horizontal axis, the maximum luminescence intensity (square blue plot and solid line) and light-emitting time ratio (triangle red plot and dashed line) on the vertical axis. The additive concentration of perylene was defined as 1.0 wt %, which exhibited the most remarkable improvement in luminescence properties. The applied voltage was an AC voltage of 5.0 V.

As shown in Figure 4a, the maximum luminescence intensity became lower and the light-emitting time ratio became larger with the increase in applied frequency. This result was similar to the result of the P3OT ECL device and the general low molecular ECL device using a ruthenium complex [8,10,13]. It is probably due to the gradual occurrence of the annihilation reaction near to the electrodes accompanying an increase in the speed of polarization reversal as the applied frequency becomes higher. Namely, the gradual generation and migration of radical cations and anions cause gradual occurrence of the annihilation reaction, which contributes to luminescence.

Luminescence was observed until the applied frequency reached 625 Hz. When AC voltage at 625 Hz was applied, an improvement of the light-emitting time ratio with a maximum, about 55 times (Figure 4b) improvement was achieved. By contrast, when an AC voltage of 10 Hz was applied, improvement of the maximum of luminescence intensity with a maximum, about 70 times (Inset of Figure 4b, 9.05 cd/m^2^) improvement was achieved. 

### 2.2. Consideration of Light-Emitting Assistance Mechanism of Perylene

The light-emitting assistance effect of perylene on the P3OT ECL device was revealed. When the ECL mechanism of P3OT on the molecular level is considered, firstly, the P3OT radical cation is generated by an electron in a thiophene ring being taken, and the P3OT radical anion is then generated by an electron being given to a thiophene ring. Usually, such electronic transfer is performed in the carbon atom, which is adjacent to a sulfur atom and separated from the alkyl chain of a side chain [14]. Then, the luminescence from the P3OT ECL device occurs by the annihilation reaction between the radical cation and the anion parts in the P3OT molecular chain accompanied by migration of radical ions or reversal of polarization. On the other hand, the perylene radical cation is generated by an electron in a benzene ring being taken, and the perylene radical anion is then generated by an electron being given to a benzene ring. Light-emitting assistance is probably due to the involvement of the perylene radical cation and anion with the redox reactions of P3OT.

To determine such a light-emitting assistance mechanism of perylene in detail, we conducted luminescence experiments under various conditions and measured the photophysical and electrochemical characteristics of both materials.

Big differences were confirmed with threshold voltage and luminescence behavior under application of DC voltage between the P3OT ECL device containing perylene and the one without perylene. These results are summarized in Table 1. Here, the voltage when the ECL device emitted luminescence of more than 0.02 cd/m^2^ luminescence intensity was defined as “the threshold voltage”.

**Table 1:** Threshold voltages under the application of AC voltage at 50 Hz and DC voltage, and the luminescence behavior when applying DC voltage from the ECL device using poly(3-octylthiophene-2,5-diyl) (P3OT) and perylene, P3OT, and perylene, respectively.

Material	P3OT and perylene	P3OT	Perylene
Threshold voltage when applying AC voltage at 50 Hz (V)	2.4	2.5	8.0
Threshold voltage when applying DC voltage (V)	2.5	7.0	10.0
Luminescence behavior when applying DC voltage	Continuous	Short	Short

In order to obtain ECL from the P3OT ECL device under the application of DC voltage, applying voltage of more than 7.0 V was required. The ECL was short luminescence of about several seconds. However, ECL was obtained by applying a lower voltage as 2.5 V when perylene was added to the emitting solution. The ECL was continuous luminescence of several minutes. These differences suggested that perylene played the role of light-emitting assistance of P3OT by moving around in the emitting solution. 

When DC voltage is applied, the annihilation reaction between radical cations and anions occurs away from electrodes, accompanied by migration of radical ions toward the opposite electrodes slightly. However, in general, the high molecular weight of the conjugated polymer hinders such a migration. Therefore, it is hard for the P3OT radical ions to migrate toward the opposite electrodes, and excessive voltage application, over the voltage that was required for the generation of the P3OT radical ions, was needed to obtain luminescence. In addition, on consideration of the result that the threshold voltage was decreased by adding perylene, it is presumed that the light-emitting assistance of perylene to the P3OT ECL device is performed by certain electronic interactions between P3OT (or P3OT radical ions) accumulated near the electrodes and the perylene (or perylene radical ions) which are moving around in the emitting solution.

**Figure 5:**
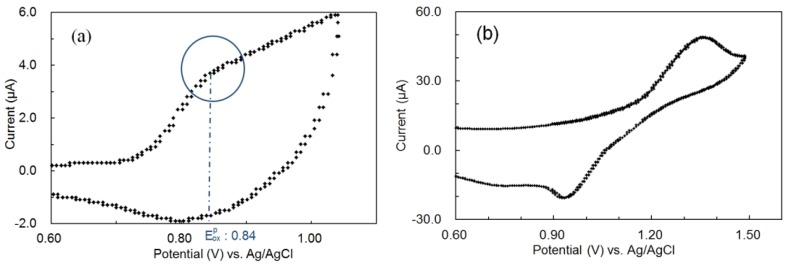
Cyclic voltammograms of (**a**) 3.5 × 10^−2^ wt % P3OT in 1,2-dichlorobenzene (DCB) containing 0.1 M tetra-butyl ammonium perchlorate (TBA) and (**b**) 1.0 × 10^−2^ M perylene in DCB containing 0.1 M TBA at a sweep rate of 20 mV/s (Working electrode: gold; Counter electrode: platinum; Reference electrode: Ag/AgCl).

In order to discuss such electronic interactions between P3OT and perylene from the energy aspect, cyclic voltammetry (CV) and ultraviolet-visible (UV-Vis) absorption spectra measurements were performed. In addition, the energy levels of both materials were estimated from these experimental results. Figure 5 shows the cyclic voltammograms of P3OT (Figure 5a) and perylene (Figure 5b) from the oxidation side, and Table 2 shows the summary of CV and UV-Vis absorption spectra measurement results. The measurement results of 9,10-diphenylanthracene (DPA) [15], pyrene [16], and naphthacene [17] are also summarized in Table 2 for comparison. 

**Table 2:** Summary of electrochemical and photophysical characteristics of focused materials measured by cyclic voltammetry (CV) and ultraviolet-visible (UV-Vis) absorption spectra measurements.

Material	$E_{ox}^{p}\;(V)$	$E_{red}^{p}\;(V)$	$E_{ox}^{1/2}\;(V)$	E_HOMO_ (eV)	E_LUMO_ (eV)	λ_onset_ (nm)	E_g_ (eV)
P3OT	+0.84	−1.33	+0.82	−4.97	−2.62	529	2.35
Perylene	+1.36	−0.93	+1.15	−5.30	−2.53	448	2.77
9,10-Diphenylanthracene (DPA)	+1.57	−1.25	+1.45	−5.60	−2.57	410	3.03
Pyrene	+1.05	No data	+0.81	−4.96	−1.35	344	3.61
Naphthacene	+1.25	No data	+1.13	−5.28	−2.72	485	2.56

In Table 2, $E_{ox}^{p\;}$ and $E_{red}^{p\;}$ represent the oxidation and reduction peak potentials, which were obtained from cyclic voltammograms, and $E_{\;ox}^{1/2}$ represents the half wave potential between peak anodic and peak cathodic potentials on the oxidation side [18]. The inflection point of the current (blue circle in Figure 5a) was seen as an oxidation peak potential regarding P3OT [19,20]. In addition, the peak cathodic potential on the oxidation side was not obtained from naphthacene. Therefore, the half wave potential between onset potential and oxidation peak potential was seen as an oxidation peak potential regarding naphthacene as reference.

The energies of the highest occupied molecular orbital (HOMO) and the lowest unoccupied molecular orbital (LUMO) can be estimated based on the energy levels of ferrocene (4.8 eV below the vacuum level) according to the following equation (1) [21,22]:

(1) $$E_{HOMO,\;LUMO}=-[(E_{ox,\;red}-E_{\;fe}^{1/2})+4.8]\;(eV)$$

where E_HOMO_ and E_LUMO_ represent the HOMO and LUMO energy levels, respectively. $E_{\;fe}^{1/2}$ is the half wave potential of ferrocene, which is 0.65 V* versus* Ag/AgCl. E_ox_ and E_red_ are the oxidation and reduction potentials, and are equal to $E_{\;ox}^{1/2}$ and $E_{red}^{1/2}$ [21,23]. In this study, there were some materials whose $E_{red}^{1/2}$ value could not be clearly determined. Therefore, the E_LUMO_ was derived from the E_HOMO_ and the optical band gap (E_g_). E_g_ was calculated from the following Equation (2) [21]:

(2) $$E_{g}=1242/\lambda_{onset}\;(eV)$$

where λ_onset_ is the longest wavelength onset of the UV-Vis absorption spectrum.

P3OT and perylene exhibited good reversible cyclic voltammograms, and the E_HOMO_ and E_LUMO_ of both materials are close. The E_HOMO_ of perylene (−5.30 eV) is lower than that of P3OT (−4.97 eV), and the E_LUMO_ of perylene (−2.53 eV) is higher than that of P3OT (−2.62 eV). The energetical stability of each radical ion is considered; the P3OT radical cation is generated by giving an electron to the perylene radical cation when the perylene radical cation is generated, and the P3OT radical anion is generated by receiving an electron from the perylene radical anion when the perylene radical anion is generated, respectively. Such an energy relationship between both materials encourages the efficient generation of P3OT radical cations and anions, and it is presumed to have greatly influenced the light-emitting assistance of perylene.

Moreover, the light-emitting assistance effect to the P3OT ECL device was not confirmed when DPA or pyrene was added instead of perylene. The E_LUMO_ of DAP is near to that of P3OT, and the E_HOMO_ of pyrene is near to that of P3OT as shown in Table 2. Thus, it is considered to be important to use materials whose HOMO and LUMO energy levels are both near to those of P3OT. On the other hand, the light-emitting assistance effect was also not confirmed when naphthacene was added, which has HOMO and LUMO energy levels near to those of P3OT. The reasoning includes the E_LUMO_ of naphthacene (−2.72 eV), which was lower than that of P3OT (−2.62 eV) as well as the electrochemical instability of naphthacene, stated previously as the peak cathodic potential on the oxidation side which here was not obtained. Therefore, we presume that it is not only the closeness of HOMO and LUMO energy levels of both materials that is related to the light-emitting assistance, but also the positional relationship of HOMO and LUMO energy levels of both materials together with the electrochemical stability of the added material that are pertinent to the light-emitting assistance.

**Figure 6:**
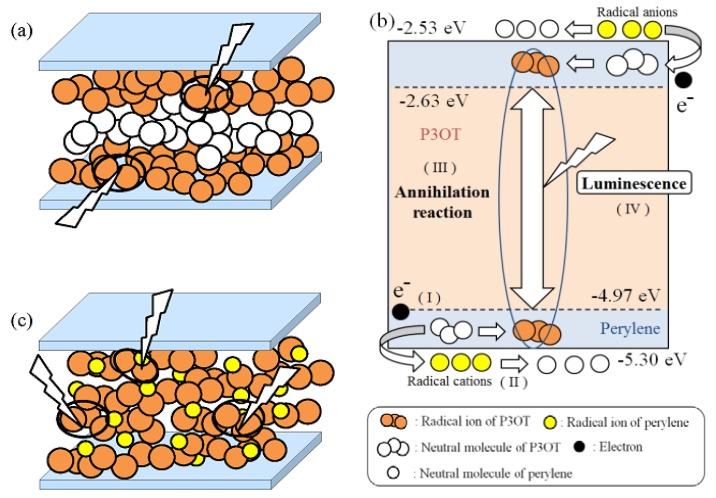
Schematic illustration of light-emitting assistance mechanism of perylene. (**a**) Light-emitting mechanism of P3OT ECL device; (**b**) Energy level diagram of P3OT and perylene; (**c**) Light-emitting mechanism of P3OT ECL device containing perylene.

Based on the above, the light-emitting assistance mechanism of perylene to the P3OT ECL device, which we constructed, is shown in Figure 6 as a schematic illustration. When AC voltage is applied to the P3OT ECL device, P3OT radical cations and anions are generated near electrodes. Because of the high molecular weight and relatively low mobility of P3OT, generated P3OT radical ions accumulate near electrodes (Figure 6a), and then the annihilation reaction occurs near the electrodes accompanied by reversal of polarization. Thus, neutral molecules of P3OT away from electrodes are probably not well utilized for the annihilation reaction. If perylene with higher mobility compared with P3OT is added here, perylene will migrate to near the center of the emitting solution under application of the same voltage. Moreover, perylene radical ions shuttle electrons to P3OT and contribute to efficient generation of P3OT radical ions because of their positional relationship of HOMO/LUMO energy levels (Figure 6b). Consequently, P3OT radical ions are generated away from electrodes, and the annihilation reaction between radical cations and anions then occurs uniformly in the emitting solution (Figure 6c). Light-emitting assistance is presumed to be conducted by such a mechanism and the luminescence properties of the P3OT ECL device are significantly improved. The light-emitting assistance effect of perylene was also confirmed when poly(3-dodecylthiophene-2,5-diyl) (P3DDT) was used instead of P3OT. P3DDT has an extended long alkyl chain with a four carbon backbone compared with P3OT, and the electrochemical and photophysical characteristics were almost identical to those of P3OT as shown in Table 3. Therefore, the light-emitting assistance of perylene to the P3DDT ECL device is thought to be conducted by the same mechanism as in the case of the P3OT ECL device. Because of a longer alkyl chain, a higher-concentrated polymer solution became available for the P3DDT ECL device. 

**Table 3:** Comparison of electrochemical and photophysical characteristics of poly(3-dodecylthiophene-2,5-diyl) (P3DDT) and P3OT.

Material	Wavelength of PL spectrum maximum (nm)	Wavelength of ECL spectrum maximum (nm)	$E_{ox}^{1/2}\;(V)$	E_HOMO_ (eV)	E_LUMO_ (eV)	λ_onset_ (nm)	E_g_ (eV)
P3DDT	568	578	+0.81	−4.96	−2.61	529	2.35
P3OT	571	577	+0.82	−4.97	−2.62	529	2.35

## 3. Experimental Section 

### 3.1. Reagents and Materials

**Figure 7:**
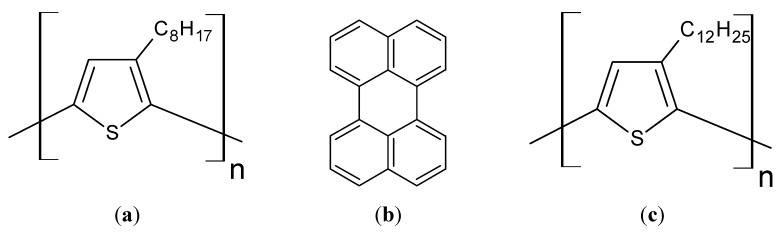
Molecular structures of (**a**) P3OT; (**b**) perylene and (**c**) P3DDT.

P3OT was used as an emitting material, and perylene (Wako Pure Chemical Industries, Ltd.) was used as a light-emitting assistant material for fabricating the ECL device in this study. Molecular structures of these materials are shown in Figure 7a,b, respectively. P3OT was synthesized by oxidative polymerization according to precedent procedures [14,24,25]. The following reagents were used for oxidative polymerization of P3OT: 3-octylthiophene (Wako); ferric chloride (Wako); chloroform (CHCl_3_, Wako); aqueous ammonia (JUNSEI CHEMICAL CO., LTD.); methanol (Wako); ethanol (Wako). P3DDT was also synthesized by oxidative polymerization using 3-dodecylthiophene (Wako) as a monomer.

DCB (99%, Wako) was used as a solvent. TBA (Tokyo Chemical Industry Co., Ltd.) was used as a supporting electrolyte and ITO glass substrate was used as an electrode.

DPA (Sigma-Aldrich Co. LLC.), pyrene (Tokyo Chem.), and naphthacene (Tokyo Chem.) were used as comparative materials with perylene in CV and UV-Vis absorption measurements.

### 3.2. Material Characterization

In order to investigate the characteristic values of synthesized P3OT and P3DDT, elemental analysis, analysis by hydrogen nuclear magnetic resonance (^1^H-NMR), and molecular weight measurement by gel chromatography (GPC) were conducted. 

#### 3.2.1. P3OT

Elemental analysis: calculation for C_12_H_18_S: C, 74.15; S, 16.50; H, 9.35%; found: C, 73.54; S, 16.53; H, 9.40%. ^1^H-NMR (300 MHz; CDCl_3_; C_12_H_18_S) δ: 7.03 (q, 1H: 1/4 at 6.99, 1/4 at 7.01, 1/4 at 7.04, and 1/4 at 7.06), 2.81 (m, 1H for HT), 2.56 (m, 1H for HH), 1.72 (m, 1H for HT), 1.61 (m, 1H for HH), 1.50–1.20 (m, 10H), 0.88 (t, 3H) [26]. The result of GPC was as follows: The number average molecular weight (M_n_), 3.03 × 10^4^; The weight average molecular weight (M_w_), 1.63 × 10^5^; The polydispersity index (M_w_/M_n_), 5.39.

#### 3.2.2. P3DDT

Elemental analysis: calculation for C_16_H_26_S: C, 76.90; S, 12.70; H, 10.40%; found: C, 77.27; S, 12.03; H, 11.76%. ^1^H-NMR (300 MHz; CDCl_3_; C_16_H_26_S) δ: 7.02 (q, 1H: 1/4 at 6.98, 1/4 at 7.01, 1/4 at 7.03, and 1/4 at 7.06), 2.80 (m, 1H for HT), 2.55 (m, 1H for HH), 1.70 (m, 1H for HT), 1.61 (m, 1H for HH), 1.50–1.20 (m, 10H), 0.87 (t, 3H) [26,27]. The result of GPC was as follows: M_n_, 2.58 × 10^4^; M_w_, 8.18 × 10^4^; M_w_/M_n_, 3.17.

### 3.3. Measurement Systems

A bipolar power supply (KIKUSUI ELECTRONICS Co., PBX40-5) was used as a voltage generator. PL and ECL spectra were measured with a spectrophotofluorometer (HITACHI, LTD., F-2000). For measurement of PL spectrum, a xenon lamp (wavelength of 350 nm) was used as excitation light. Both spectra were measured by the form of the ECL device. Time variation of luminescence intensity was measured by a luminance colorimeter (TOPCON Co., Ltd., BM-7) under a nitrogen atmosphere.

CV measurements were executed using gold as working electrode, platinum as counter electrode, and Ag/AgCl as reference electrode. Potentio-galvano stat (HOKUTO DENKO Co., HAB-151) was used as applied voltage, and its sweep rate was set at 20 mV/s. The sample solution was prepared by dissolving 3.5 × 10^−2^ wt % polymer material or 1.0 × 10^−2^ M low molecular material in DCB containing 2.5 wt % (0.1 M) TBA. Before carrying out the measurements, argon was passed through the sample solution to replace traces of oxygen.

UV-VIS absorption spectra were measured by a spectrophotometer (HITACHI, LTD., U3310) using a sample solution which consisted of about 3.0 × 10^−3^ wt % sample material dissolved in CHCl_3_. 

All measurements were performed at 26 degrees Celsius room temperature. 

## 4. Conclusions 

Perylene exhibited a light-emitting assistance effect to the polymer ECL device using P3OT, whereby its luminescence properties were improved. We investigated the improvement in luminescence properties quantitatively, and found that it became the most notable when 1.0 wt % perylene was added to the ECL device using 3.0 wt % P3OT. At that point, improvements of about 12 times of the maximum luminescence intensity and about 23 times of the light-emitting time ratio compared to that of the P3OT ECL device were confirmed. Moreover, by changing the applied frequency improvements of the light-emitting time ratio of about 70 times and of the light-emitting time ratio of about 55 times were achieved. 

The measurement results of PL and ECL spectra indicated that ECL was occurring only from excited-state P3OT molecules and that perylene plays the role of light-emitting assistant of P3OT. We presumed that the light-emitting assistance by perylene is achieved based on the difference of mobility in the emitting solution and the positional relationship of energy levels between P3OT and perylene. The light-emitting assistance is presumed to be conducted by perylene radical ions shuttling electrons to P3OT while moving around in the emitting solution. Moreover, light-emitting assistance effect was confirmed even if P3DDT, which has almost identical photophysical and electrochemical characteristics to those of P3OT, was used instead of P3OT. Therefore, in future study, realization of the ECL device, which has more outstanding luminescence properties is expected by exploring the polymer ECL device containing low molecular material. This has HOMO and LUMO energy levels corresponding to those of polymer material such as the P3OT ECL device containing perylene.
